# The Role of Ultrasonographic Assessment of Optic Nerve Sheath Diameter in Prediction of Sepsis—Associated Encephalopathy: Prospective Observational Study

**DOI:** 10.1007/s12028-024-02187-9

**Published:** 2025-01-15

**Authors:** Sherif M. S. Mowafy, Hany Bauiomy, Neveen A. Kohaf, Shereen E. Abd Ellatif

**Affiliations:** 1 Department of Anesthesia, Intensive Care, and Pain Management, Faculty of Medicine, Zagazig University, Zagazig, Egypt; 2 Department of Anesthesia and Intensive Care, Faculty of Medicine, Benha University, Benha, Egypt; 3 Department of Clinical Pharmacy, Faculty of Pharmacy (Girls), Al-Azhar University, Cairo, Egypt; 4 Department of Clinical Pharmacy, Faculty of Clinical Pharmacy, Al-Baha University, Al-Baha, Saudi Arabia

**Keywords:** Optic nerve sheath diameter, Sepsis, Sepsis-associated encephalopathy, Brain dysfunction

## Abstract

**Background:**

Ultrasonographic optic nerve sheath diameter (ONSD) is a satisfactory noninvasive intracranial pressure (ICP) monitoring test. Our aim was to evaluate ONSD as an objective screening tool to predict and diagnose ICP changes early in sepsis-associated encephalopathy (SAE).

**Methods:**

Our prospective observational study was conducted on patients with sepsis, and after intensive care unit (ICU) admission, the time to diagnose SAE was recorded, and patients were divided into a non-SAE group including conscious patients with sepsis and a SAE group including patients with sepsis with acute onset of disturbed conscious level. ONSD was measured within 24 h of ICU admission for all patients and then every other day for up to 10 consecutive days until ICU discharge or death. The primary outcome was to compare ONSD measurements of both groups to find if there was a correlation between ONSD and SAE occurrence.

**Results:**

Eighty-nine patients with sepsis were divided into a non-SAE group (*n* = 45) and an SAE group (*n* = 44). ONSD showed a statistically significant difference at day 0 and a highly significant difference at days 2, 4, 6, 8, and 10. Day 2 ONSD had the best accuracy for predicting SAE, with a cutoff > 5.2 mm (sensitivity of 93.2%, specificity of 100%), a statistically positive correlation with the Sequential Organ Failure Assessment score (*r* = 0.485, *P* < 0.001) and ICU length of stay (*r* = 0.238, *P* < 0.001), and a statistically significant wider in patients who died compared to those who survived (*P* < 0.001).

**Conclusions:**

ONSD could be an objective screening method for early diagnosis of SAE, with a cutoff > 5.2 mm.

*Trial registration*

NCT05849831 (https://clinicaltrials.gov/study/NCT05849831).

## Introduction

Sepsis is a life-threatening organ dysfunction resulting from a dysregulated body response to an infection and has become a leading cause of morbidity and mortality worldwide in adult critically ill patients. The brain shows high vulnerability to the inflammatory storm associated with infection, and it may be the first organ to show signs of life-threatening organ dysfunction caused by infection. This brain dysfunction is known as sepsis-associated encephalopathy (SAE) [1–3]. Sepsis-associated brain dysfunction is considered the most common type of encephalopathy seen in the intensive care unit (ICU), and it may occur in up to 70% of septic patients as well as significantly increase mortality [4]. SAE is defined as a life-threatening acute diffuse brain dysfunction due to infection outside the central nervous system (CNS) and is mostly caused by the inflammatory storm. It varies from delirium or confusion, seizure or focal neurological signs, and diffuse or multifocal neurological deficits to stupor or coma with no other diagnoses describing the patient’s neurological status [4]. It is a complex syndrome with unclear pathophysiology. The possible causes could incorporate neuroinflammation, excitotoxicity, compromised cerebral autoregulation, and cerebral ischemia [5–7].

Elevated intracranial pressure (ICP) is one of the main causes of disturbed consciousness, and the pathophysiological alterations that occur in patients with sepsis could increase the ICP, which may in turn decrease cerebral perfusion and lead to brain edema and brain damage and compromise the patients’ outcomes [8, 9]. In sepsis, the overactivated inflammatory immune response and the markedly released cytokines trigger cerebrovascular endothelial cell dysfunction and damage, increasing blood–brain barrier (BBB) permeability, which leads to brain edema that contributes significantly to SAE pathophysiology [10]. In addition to sepsis-induced BBB damage, other factors such as fever, increased thoracic and abdominal pressure and excessive fluid resuscitation for sepsis management may worsen the brain edema, resulting in increased ICP in these patients [8, 9]. Therefore, in addition to the sepsis management measures, early detection of increased ICP is of paramount importance for timely intervention and improved prognosis. However, direct invasive ICP monitoring is not routinely recommended in nontraumatic coma patients and septic patients either because it has serious complications, including the risks of CNS hemorrhage and infection, or because it is contraindicated due to the presence of coagulopathy in septic patients [9, 11]; so a reliable noninvasive ICP monitoring method is highly recommended.

Bedside ultrasonographic assessment of the optic nerve sheath diameter (ONSD) offers a satisfactory simple noninvasive ICP monitoring test, and it has been reported to be strongly correlated with both invasive ICP measurements [12–16] and radiographic cerebral edema as identified by either computed tomography [17, 18] or magnetic resonance imaging [9, 19], with numerous studies documenting its clinical benefits as an estimate of the ICP. However, most of these studies focused on patients with traumatic brain injury, cerebral hemorrhage, or infarction [12–16].

Considering that brain edema and increased ICP are common pathophysiological alterations in patients with SAE, consequently, it is feasible to use ultrasonographic ONSD to evaluate ICP and detect brain dysfunction early in these patients. However, there is little published literature on ONSD use in SAE, and its clinical benefits as a monitoring method in these patients require further investigation. Moreover, SAE diagnosis is still challenging, relying on the exclusion of other causes of brain dysfunction with the need for an objective screening method. We hypothesized that ONSD could have a role as an objective screening tool to predict and diagnose ICP changes early in septic patients.

The aim of this study was to compare the ONSD measurements of patients with SAE to those of patients without SAE in a trial to find if there is a correlation between ONSD measurements and the occurrence of SAE and to assess ONSD’s role in SAE prediction.

## Methods

### Study Population and Design

This prospective observational study was conducted at Zagazig University Hospitals and Benha University Hospitals from May 2023 to May 2024 after being reviewed and approved by the research ethics committees of both the Faculty of Medicine, Zagazig University (ZU-IRB#10597-30/4-2023), and the Faculty of Medicine, Benha University (RC4-11-2023) (ClinicalTrials.gov identifier: NCT05849831). Written informed consent from all participants or their legal guardians was obtained.

Eighty-nine patients admitted to the surgical ICUs in Zagazig University Hospitals and Benha University Hospitals with a diagnosis of sepsis aged between 21 and 70 years old were enrolled in our study. Zagazig University Hospitals and Benha University Hospitals are tertiary care teaching hospitals with surgical ICUs dedicated to the care of critically ill patients from neurosurgical procedures and general, vascular, gastrointestinal, trauma, orthopedic, and obstetric surgeries.

Patients who refused to participate in this study and patients with a history of ocular trauma or surgery, ocular wound, conjunctival edema, or orbital edema; patients with difficulty in obtaining an ONSD image, brain trauma, cerebrovascular accident, CNS infection, and any previous neurological procedure; and those who developed any other type of encephalopathy other than SAE were excluded from this study. The withdrawal criteria after enrollment involved patients who required deep sedation as well as those who were discharged before the fifth ICU day because they were either improved, died, or discharged against medical advice. The data of these patients were not included in the final analysis.

Upon ICU admission, all patients were subjected to careful history taking, thorough clinical examination, and standard laboratory workup. Patients diagnosed with sepsis were included in our study, and their clinical illness severity was assessed by using the Acute Physiology and Chronic Health Evaluation II (APACHE II) on admission, whereas the Sequential Organ Failure Assessment (SOFA) score, which represents the severity of organ dysfunction due to sepsis, was calculated daily. The third international sepsis definition and its appropriate diagnostic criteria were used for diagnosing sepsis and septic shock. Sepsis was defined as a life-threatening organ dysfunction initiated by a dysregulated host reaction to an infection. It is suspected in acutely deteriorating patients in whom there is clinical evidence or strong suspicion of infection. Septic shock was defined by the requirement for vasopressors or vasoactive medication to maintain a mean arterial blood pressure of 65 mm Hg or higher after adequate fluid resuscitation, with the presence of a high lactate level (> 2 mmol/L) [2].

All enrolled study participants underwent ultrasonographic assessment of ONSD within 24 h of ICU admission (day 0, which is the day of ICU admission).

### Ultrasonographic Measurement of ONSD

Ocular ultrasonography was performed by two skilled examiners, one in each university hospital who have the same level of experience (more than 2 years’ experience in sonography and performed more than 100 ultrasonographic ONSD measurements previously) and used the same technique to perform ultrasonographic measurements of ONSD in our study. After placing the patients in a supine position with the head of the bed 30 degrees above the horizontal line, a layer of ultrasound gel was applied over the closed upper eyelid, and the liner high-frequency probe (7–12 MHz) of the ultrasound machine (SonoSite M-Turbo and GE Logiq E machines were used in Zagazig University Hospitals and Benha University Hospitals, respectively) was placed on the eyelid temporal area with the holding hand resting on the patient forehead to prevent unnecessary pressure being exerted on the eye. The probe was then adjusted to a suitable angle to display the entry of the optic nerve into the globe. ONSD was measured 3 mm behind the globe in the transverse plane perpendicular to the optic nerve. For each eye, one measurement was obtained, and the stated ONSD corresponds to the mean of the two values obtained for each patient. An average ONSD greater than 5 mm was considered abnormal, and high ICP should be assumed [20, 21].

After admission, all enrolled patients were assessed daily for any SAE manifestations, and the time from ICU admission until the onset of SAE was recorded. SAE was diagnosed by the combination of extracranial infection with clinical signs of neurological dysfunction. SAE clinical manifestations include impairment of awareness, which ranges from delirium to coma [4]. Other factors producing mental changes and encephalopathy were excluded, as well as brain computed tomography already done to evaluate the disturbed consciousness state. Therefore, the managing ICU team decided to diagnose patients with SAE, and the time from ICU admission to the onset of SAE was documented.

The Confusion Assessment Method for the Intensive Care Unit (CAM-ICU) was used for diagnosing delirium (delirium was defined as any positive CAM-ICU examination), and the Glasgow Coma Scale (GCS) was used for monitoring coma. SAE was diagnosed as GCS < 15 points and/or delirium (positive CAM-ICU) [4]. GCS and CAM-ICU examinations were done for all enrolled patients who did not require sedation and were performed for patients on sedative and analgesic drugs if the Richmond Agitation Sedation Score (RASS) was equal to or greater than − 2. Patients who required analgesic and sedative drugs resulting in RASS − 3 to − 5, defined as deep sedation, were not included in the final analysis.

Accordingly, the enrolled patients were divided into two groups:

non-SAE group, fully conscious patients with sepsis on ICU admission;

SAE group, patients with sepsis on ICU admission with acute onset of disturbed consciousness during their ICU stay.

ONSD measurements, SOFA score calculation, and GCS and CAM-ICU assessment were performed for all enrolled patients in both groups and recorded every other day for up to 10 consecutive days until ICU discharge or death.

### Sample Size Calculation

Our study aimed to evaluate ONSD’s role as an objective screening tool and to find out the cutoff for ONSD to predict and diagnose ICP changes early in patients with SAE. To be able to predict SAE early, we supposed that ONSD on day 0 of ICU admission could have a significant role, and so from our pilot study of 10 patients (5 in each group), we found that the mean ± SD of ONSD on day 0 of ICU admission among the non-SAE group was 5.12 ± 0.16 mm and that among SAE group patients, it was 5.2 ± 0.1 mm, so the sample size was calculated using an open epi program to be 88 patients (44 in each group), with a confidence level of 95% and power test of 80%. The pilot study data were not included in the study’s final analysis.

### Data Collection

Patient data, including age, sex, body mass index, APACHE II score, the time to diagnose SAE (days), ICU length of stay (ICU-LOS), and ICU mortality, were recorded. ONSD measurements and SOFA score assessments were recorded every other day for up to 10 consecutive days until ICU discharge or death.

The primary outcome of this study was to assess ONSD’s role as an objective screening tool and to find out its cutoff for SAE prediction by comparing the ONSD measurements of patients with SAE patients to those of patients without SAE. The secondary outcomes were to examine the association of ONSD and the SOFA score with ICU mortality in our included critically ill patients with sepsis.

### Statistical Analysis

Statistical analysis was done by SPSS v27 (IBM, Armonk, NY). The Shapiro–Wilks test and histograms were used to evaluate the normality of the distribution of data. Quantitative parametric data were presented as mean and standard deviation and were analyzed using unpaired Student’s *t*-test. Qualitative variables were presented as frequency and percentage and analyzed using the χ^2^ test or Fisher’s exact test when appropriate. Pearson correlation was applied to evaluate the correlation between variables. Receiver operating characteristic (ROC) curve analysis was used to evaluate the role of ONSD (mm) for prediction of SAE. A two-tailed *P* value < 0.05 was considered statistically significant.

## Results

**Fig. 1:**
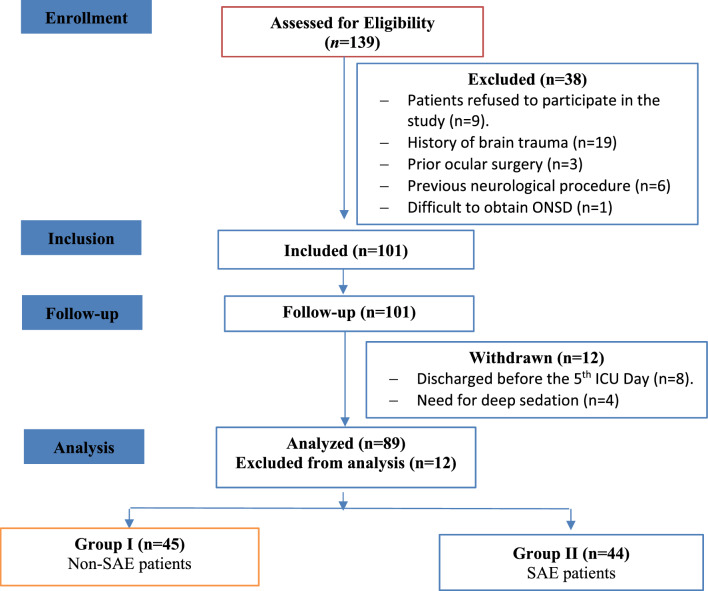
STROBE flowchart of the studied patients. ICU intensive care unit, ONSD optic nerve sheath diameter, SAE sepsis-associated encephalopathy, STROBE strengthening the reporting of observational studies in epidemiology.

In this study, 139 patients were assessed for eligibility, and 38 patients did not meet our inclusion criteria. The remaining 101 patients were consented and enrolled in our study. Later, during the follow-up period, 12 patients were withdrawn. The final study sample was 89 patients who matched our inclusion criteria, completed the follow-up period, and were classified into two groups: group I (non-SAE: *n* = 45) and group II (SAE: *n* = 44) (Fig. 1). Among patients with SAE, the time to SAE diagnosis after enrollment was 3.09 ± 1.48 days.

The patients’ characteristics, including age, sex, and body mass index, were statistically nonsignificantly different between the two studied groups, whereas the APACHE II score, ICU-LOS (days), and ICU mortality were statistically significantly higher in the SAE group compared to the non-SAE group (Table 1). Chest infection was found to be the most common source of sepsis in both groups (55.56% and 47.73% in SAE and non-SAE groups, respectively), and there was no statistically significant difference between the two groups regarding the main source of infection (Table 1).

**Table 1:** Clinical characteristics of the studied groups

Characteristics	Group I (*n* = 45)	Group II (*n* = 44)	*P* value
Age (years), mean ± SD	50.69 ± 9.69	49.93 ± 8.97	0.703
Sex, *n* (%)			
Male	34 (75.56)	30 (68.18)	0.439
Female	11 (24.44)	14 (31.82)	
BMI, mean ± SD	28.04 ± 4.32	28.43 ± 5.35	0.708
APACHE II score, mean ± SD	12.87 ± 2.48	14.82 ± 5.01^a^	0.022
ICU-LOS (days), mean ± SD	12.6 ± 3.5	15.05 ± 3.82^b^	0.002
ICU mortality, *n* (%)			
Alive	33 (73.33)	23 (52.27)	0.04
Dead	12 (26.67)	21 (47.73)^b^	
The main source of infection, *n* (%)			
Chest	25 (55.56)	21 (47.73)	0.8664
Intraabdominal	8 (17.78)	9 (20.45)	
Skin and soft tissue	5 (11.11)	7 (15.9)	
UTI	7 (15.56)	7 (15.9)	

Group I consisted of patients without SAE, and group II consisted of patients with SAE

APACHE II, Acute Physiology and Chronic Health Evaluation II, BMI, body mass index, ICU, intensive care unit, ICU-LOS, intensive care unit length of stay, SAE, sepsis-associated encephalopathy, UTI, urinary tract infection

*P* < 0.05 is significant; *P* < 0.001 is a highly significant difference

^a^APACHE II score was significantly high in patients with SAE

^b^ICU-LOS and mortality were significantly high in patients with SAE

Comparing the ONSD measurements of patients with SAE to those of patients without SAE showed a statistically significant difference between the two studied groups at day 0. Also, a statistically high significant difference was found at all other times of measurement (on days 2, 4, 6, 8, and 10) with increased ONSD measurements in patients with SAE (Table 2).

**Table 2:** ONSD at different times of measurement of the studied groups

	Group I (*n* = 45), mm	Group II (*n* = 44), mm	*P* value
Day 0	4.72 ± 0.29	4.96 ± 0.66^a^	0.035
Day 2	4.69 ± 0.34	5.84 ± 0.36^b^	< 0.001
Day 4	4.69 ± 0.31	5.94 ± 0.3^b^	< 0.001
Day 6	4.6 ± 0.25	5.66 ± 0.31^b^	< 0.001
Day 8	4.76 ± 0.35	5.6 ± 0.35^b^	< 0.001
Day 10	4.53 ± 0.24	5.62 ± 0.32^b^	< 0.001

Data were expressed as mean ± SD. Group I consisted of patients without SAE, and group II consisted of patients with SAE. Day 0 is the day of intensive care unit admission

ONSD, optic nerve sheath diameter, SAE, sepsis-associated encephalopathy

*P* < 0.05 is significant; *P* < 0.001 is a highly significant difference

^a^ONSD was significantly wider in patients with SAE at day 0

^b^ONSD was significantly increased in patients with SAE at days 2, 4, 6, 8, and 10

Although there was a statistically insignificant difference in the SOFA score evaluated in our study between the studied groups at day 0, it was significantly increased in patients with SAE with a statistically high significant difference when compared to non-SAE patients at day 2, 4, 6, 8, and 10 (Table 3).

**Table 3:** SOFA score at different times of measurement of the studied groups

	Group I (*n* = 45)	Group II (*n* = 44)	*P* value
Day 0	8.13 ± 2.29	8.98 ± 2.77	0.121
Day 2	7.13 ± 1.95	10.02 ± 3.12^a^	< 0.001
Day 4	6.09 ± 1.76	12.2 ± 2.18^a^	< 0.001
Day 6	5.4 ± 2.31	13.55 ± 3.19^a^	< 0.001
Day 8	4.81 ± 2.42	12.47 ± 3.51^a^	< 0.001
Day 10	3.31 ± 1.41	10.74 ± 2.53^a^	< 0.001

Data were expressed as mean ± SD. Group I consisted of patients without SAE, and group II consisted of patients with SAE. Day 0 is the day of intensive care unit admission

SAE, sepsis-associated encephalopathy, SOFA, Sequential Organ Failure Assessment

*P* < 0.05 is significant; *P* < 0.001 is a highly significant difference

^a^SOFA scores were significantly high in patients with SAE on days 2, 4, 6, 8, and 10

**Fig. 2:**
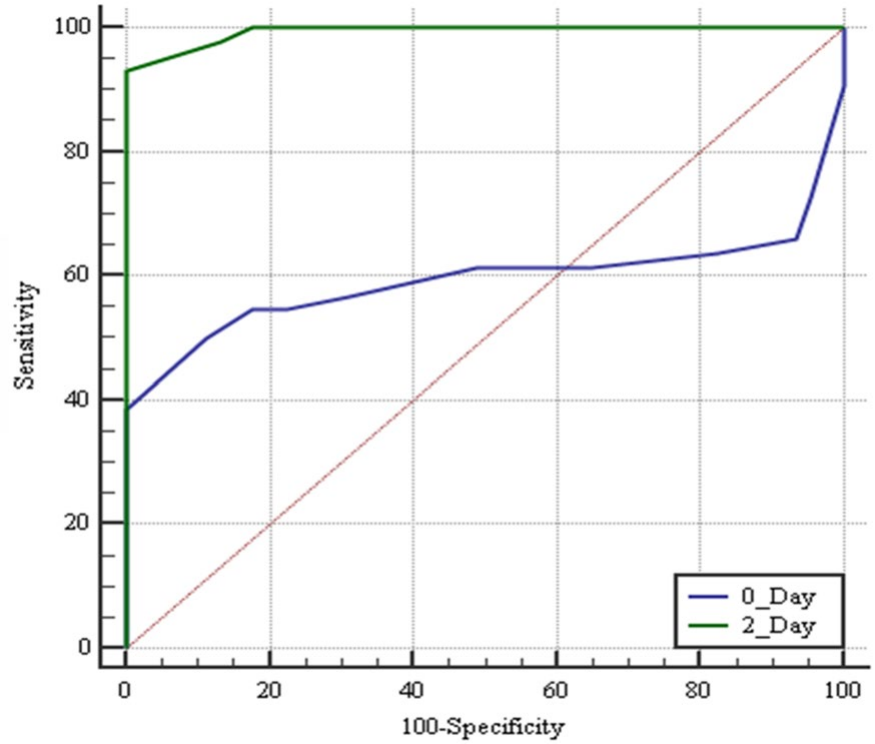
ROC analysis of ONSD at days 0 and 2. The ROC analysis results of ONSD measurements (mm) at day 0 and day 2 for predicting SAE showed that ONSD measured at day 2 had the best accuracy for predicting SAE with a cutoff value > 5.2 mm, a sensitivity of 93.2%, and a specificity of 100%. ONSD optic nerve sheath diameter, ROC receiver operating characteristic, SAE sepsis-associated encephalopathy.

**Fig. 3:**
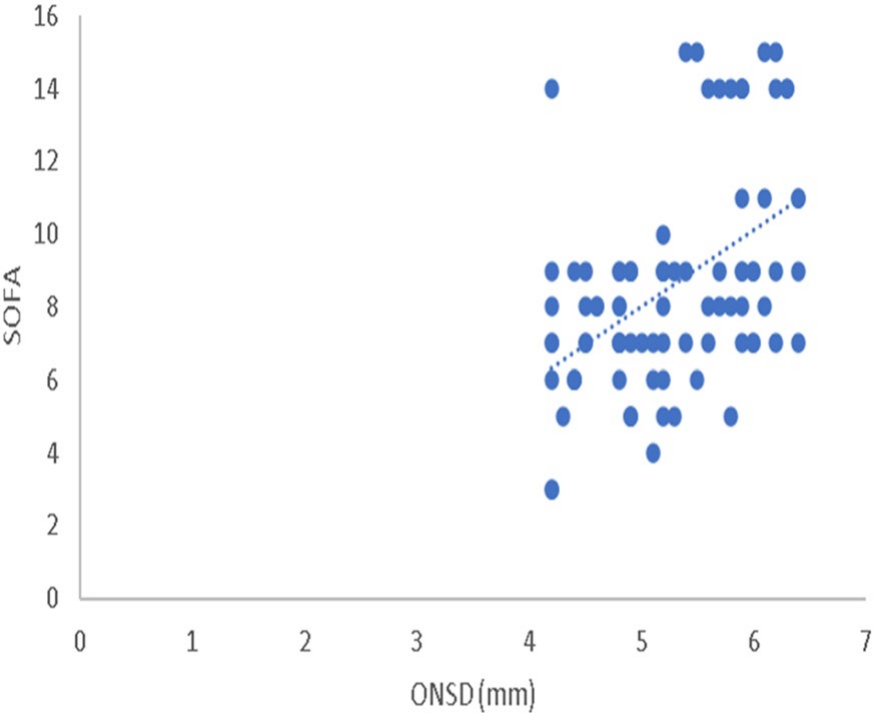
Correlation between ONSD and SOFA score on day 2. ONSD measurements on day 2 showed statistically positive correlation with SOFA score assessments (*r* = 0.485, *P* < 0.001). ONSD optic nerve sheath diameter, SOFA Sequential Organ Failure Assessment.

**Fig. 4:**
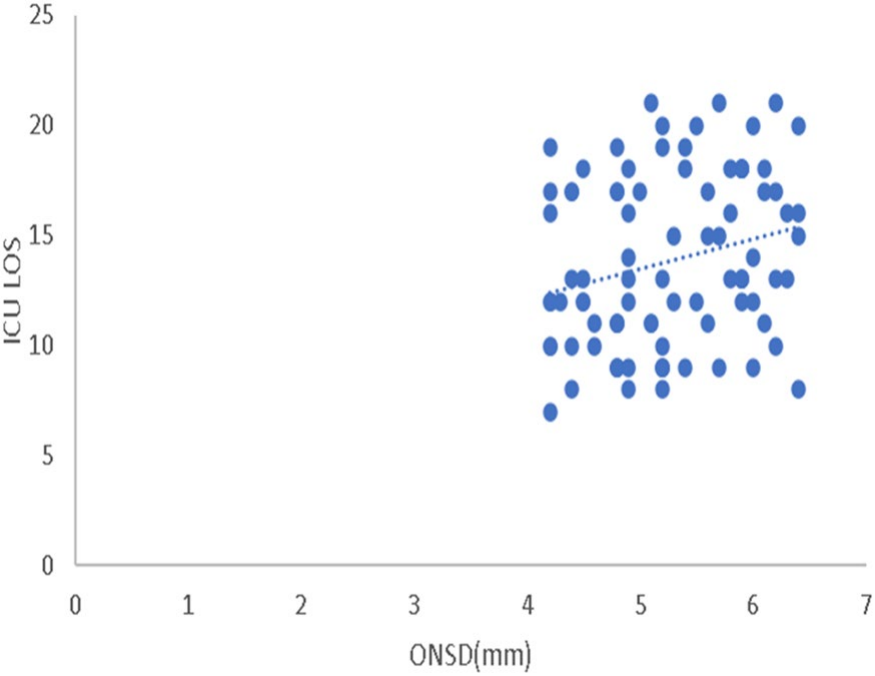
Correlation between ONSD and ICU-LOS on day 2. ONSD measurements on day 2 showed statistically positive correlation with ICU-LOS (*r* = 0.238, *P* < 0.001). ICU-LOS intensive care unit length of stay, ONSD optic nerve sheath diameter.

**Fig. 5:**
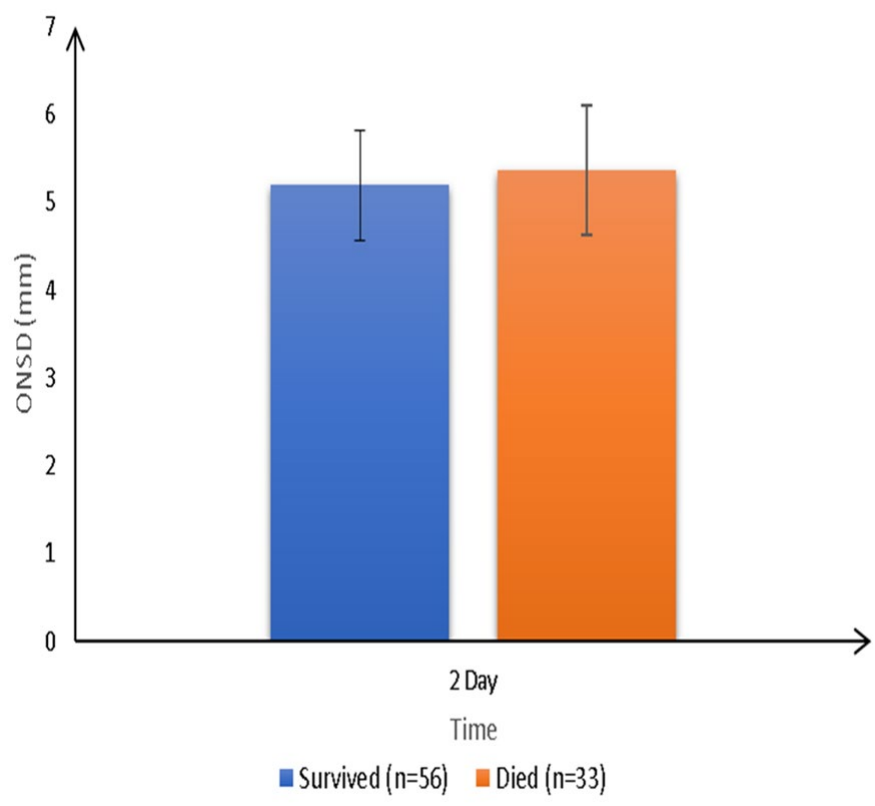
Comparison of ONSD measurements on day 2 between surviving and dead patients. ONSD measurements on day 2 were statistically significant wider in patients who died (*n* = 33) compared to patients who survived (*n* = 56) (5.84 ± 0.36 mm vs. 4.69 ± 0.34 mm, respectively; *P* < 0.001). ONSD optic nerve sheath diameter.

The ROC analysis results of ONSD measurements on day 0 and day 2 for predicting SAE showed that ONSD measured at day 2 had the best accuracy for predicting SAE, with a cutoff value > 5.2 mm, a sensitivity of 93.2%, and a specificity of 100% (Fig. 2, Table 4). Also, ONSD measurements at day 2 showed a statistically positive correlation with both the SOFA score assessments (*r* = 0.485, *P* < 0.001) (Fig. 3) and the ICU-LOS (*r* = 0.238, *P* < 0.001) (Fig. 4). Additionally, ONSD measurements at day 2 were statistically significantly wider in patients who died (*n* = 33) compared to those who survived (*n* = 56) (5.84 ± 0.36 mm vs. 4.69 ± 0.34 mm, respectively; *P* < 0.001) (Fig. 5).

**Table 4:** ONSD role in prediction of SAE

Variable	Cutoff, mm	Sensitivity, %	Specificity, %	PPV	NPV	AUC	*P* value
Day 0	> 5.1	50	88.9	81.5	64.5	0.593	0.178
Day 2	> 5.2	93.2	100	100	93.7	0.993	< 0.001

AUC, area under the curve, NPV, negative predictive value, ONSD, optic nerve sheath diameter, PPV positive predictive value, SAE, sepsis-associated encephalopathy

*P* < 0.05 is significant; *P* < 0.001 is a highly significant difference

## Discussion

SAE is considered the most common encephalopathy in surgical ICUs and is frequently reported with adverse clinical outcomes [4, 22]. Currently, there are no specific diagnostic criteria, and SAE diagnosis is often delayed and remains challenging depending on the exclusion of other causes of brain injury [23–26]. Therefore, in clinical practice, there is an urgent need for a simple noninvasive method with reasonable sensitivity and specificity for early prediction and diagnosis of SAE. This study aimed to evaluate the role of ONSD as a simple objective screening tool to predict and diagnose ICP changes early in patients with SAE. Our results found that the time to diagnose SAE after ICU admission was 3.09 ± 1.48 days and that the ONSD of patients with SAE was significantly wider than that of patients without SAE at all times of measurements. ONSD on day 2 showed the best accuracy for early SAE prediction, with a cutoff value > 5.2 mm (sensitivity of 93.2%, specificity of 100%, area under the curve [AUC] 0.993).

Pfister et al. found that 47% of patients with sepsis had ICP > 15 mm Hg [8]. Salahuddin et al. found that nontraumatic radiographic cerebral edema in coma patients was often due to SAE [27]. Luo et al. reported elevated ICP as an independent risk factor for SAE development [28]. Consequently, evaluating ICP could be beneficial in SAE diagnosis. We opted to use bedside ultrasonographic assessment of ONSD in our study because it is a simple satisfactory noninvasive ICP monitoring test, it is a sensitive and specific predictor of cerebral edema, and it is strongly correlated with invasive ICP measurements [12–16]. In their meta-analysis, Dubourg et al. documented that the ultrasonographic ONSD had a good level of diagnostic accuracy for intracranial hypertension, with a sensitivity of 90%, a specificity of 85%, and an AUC of 0.94 [29]. Additionally, the association between elevated ICP and SAE has been examined in many studies [9, 11, 28]. Therefore, ONSD offers a good opportunity for early recognition of the first signs of sepsis brain dysfunction.

In an animal study on sepsis rabbits, Wang et al. [30] found that ONSD was significantly wider in the SAE group than the control group, and ONSD changes were positively correlated with the brain injury biomarkers, including S100B, neuro-specific enolase (NSE), and myeloperoxidase (MPO), at 6, 12, and 24 h, respectively. They concluded that ONSD was helpful in SAE diagnosis, with the best predictive value at 24 h after modeling [30]. Similar findings in human studies were demonstrated by Czempik et al. [9] in their preliminary report investigating the ONSD of ten patients with septic shock as an SAE screening tool, setting the ONSD upper limit at 5.7 mm. They found that 40% of their patients had ONSD above the upper limit on the first day of examination and 70% of the patients had ONSD above 5.5 mm on the first day of examination reporting that borderline or mildly elevated ONSD in patients with septic shock could be a sign of SAE [9]. In their observation on 90 patients with sepsis, Yang et al. documented significantly wider ONSD in patients with SAE compared to both patients without SAE patients and SAE recovery patients, with the best predictive ONSD value for SAE recognition being ≥ 5.5 mm (sensitivity 80.4%, specificity 83.5%, and AUC 0.894) [11]. Recently, in a cohort of 123 patients with sepsis, Luo et al. [28] investigated the association between ONSD and the SAE incidence, reporting that the median time to SAE diagnosis after enrollment was 3.9 ± 2.7 days and the incidence of SAE was increased in patients with higher values of both the first and maximum ONSD measured values (ONSD 0 and ONSD max, respectively). Additionally, ONSD 0 and ONSD max were significantly wider in patients with SAE than those in patients without SAE, confirming that elevated ONSD values as an independent SAE risk factor and ONSD 0 and ONSD max cutoff values of 5.4 and 5.8 mm, respectively, can be used to predict SAE (for ONSD 0, sensitivity was 84.5%, specificity was 64.6%, and AUC was 0.801; for ONSD max, sensitivity 74.1%, specificity 81.5%, and AUC was 0.829) [28].

Although various studies documented high ONSD accuracy in diagnosing increased ICP [12–16], optimal ONSD cutoff levels diagnosing increased ICP differ extensively among the studies. Some studies suggested an optimal cutoff level of 5–5.5 mm [31], whereas others postulated higher cutoff levels between 5.6 and 6.1 mm [32]. In their meta-analysis, Berhanu et al. found that the optimal ONSD cutoff levels were between 4.1 and 7.2 mm and that higher cutoff levels of 5.6–6.3 mm compared to lower levels of 4.9–5.5 mm seemed to significantly increase the specificity, with similar sensitivity between both cutoff levels [33]. We found that ONSD measured on day 2 after ICU admission with a cutoff value > 5.2 mm had the best accuracy for early SAE prediction. Yang et al. [11] demonstrated that the best predictive ONSD value measured within 24 h of admission was ≥ 5.5 mm, and Luo et al. [28] recommend a cutoff value of 5.4 mm for the first measured ONSD within 6 h of admission to predict SAE. Although the available studies and this study were constructed based on finding the best predictive ONSD cutoff level to detect patients with SAE early, suggesting different cutoff levels, they agreed that implementing ONSD measurement in the diagnostic model of SAE could maximize early recognition of these patients.

Although several research pieces encourage ultrasonographic ONSD assessment in patients with sepsis, key concerns over its reproducibility, observers’ variations, and measurement accuracy have been raised because all sonographic measurements are operator dependent, and there is a wide range of normal ONSD cutoff levels. Numerous authors previously reported its high level of interobserver and intraobserver reliability. Ballantyne et al. [34] assessed the observer variations in ONSD sonographic measurement of normal adult volunteers with three examiners (one consultant radiologist and two trainees). They reported it to be a reproducible method with low interobserver and intraobserver variability, stressing the importance of standardization of the examination method [34]. Also, Bauerle et al. reported a very high level of intraobserver reliability, reaching 0.92 to 0.97 [35]. Additionally, Wang et al. [36] conducted an investigation with two observers; one observer had 8 years of ultrasonography experience, whereas the other observer was a novice resident who received only 1 week of training in transorbital ultrasonography. They found high interobserver and intraobserver reliability, confirming ONSD sonographic assessment as a highly reproducible and reliable method for ICP evaluation [36]. In concordance with these studies, Kc et al. documented high intraobserver reliability and excellent correlation of interobserver reliability between the two observers [37]. Therefore, ocular ultrasound for ONSD measurement with the standard technique has significant interobserver and intraobserver agreement, confirming its reproducibility and reliability.

In this study, for diagnosing sepsis, the third international definition and appropriate diagnostic criteria, including the SOFA score, were used [2]. The SOFA score is the most common score used and is reported by many studies to have good diagnostic and prognostic predictive value in patients with sepsis [38]. Our research demonstrated significantly high SOFA scores in patients with SAE compared to patients without SAE as well as significantly high APACHE II scores in patients with SAE. Both scores are main indicators of the severity of a patient’s condition, indicating that patients with SAE are more severely ill than patients without SAE [39]. However, their validity to diagnose SAE is still unclear [40, 41]. Patients with higher SOFA and APACHE II scores may be more likely to have SAE, but this may be a biased conclusion because it is worth mentioning that GCS assessment is one of the elements of the SOFA and APACHE II scores, and the diagnosis of SAE is defined by a GCS score < 15.

Patients with SAE tend to have higher ICU-LOS and mortality than those with sepsis alone. Eidelman et al. [42] found that the occurrence of SAE increased hospital mortality from 16% when the GCS score was 15 to 63% when the GCS score was between 3 and 8. Similar conclusions were reported by various studies linking SAE to increased ICU stay and higher short-term mortality [22, 26, 43–45]. Our study also demonstrated a significantly extended ICU stay and increased ICU mortality in patients with SAE.

In this study, ONSD measurements of patients with SAE on day 2 showed a positive correlation with both the SOFA score assessments (*r* = 0.485, *P* < 0.001) and the ICU-LOS (*r* = 0.238, *P* < 0.001), indicating that the wider the ONSD, the more severely ill the patients. In their report, Czempik et al. found no correlation between ONSD and the SOFA score, which could be explained by the different included patients because they investigated only ten patients with septic shock in a mixed ICU, and they used a higher limit of ONSD to diagnose increased ICP (5.7 mm versus 5 mm in our study) [9]. In the present study, wider ONSD was observed in patients who died compared to patients who survived, which is in line with the findings by Yang et al., who demonstrated that patients who died of SAE had slightly wider ONSD than surviving patients [11].

Our results documented that ONSD was helpful in early identification of increased ICP in patients with sepsis and that a wider ONSD reported in patients with SAE could be an early prediction of SAE occurrence, with a cutoff > 5.2 mm. High suspicion of SAE should arise in patients with sepsis with any mental status alterations. ONSD measurement implementation as a screening tool in the diagnostic model could maximize early identification of these patients and stratify the decisions of escalating and individualizing treatment with aggressive measures to optimize cerebral perfusion pressure. Therefore, future studies with efforts to include populations from different regions and different ethnic groups and using standardized methods to assess and implement ONSD measurement in the sepsis management protocol are highly required.

However, this study has some limitations. Firstly, studying SAE is challenging because there are no specific diagnostic criteria, and it remains a diagnosis of exclusion. Although we used a GCS score < 15 and a positive CAM-ICU result, which are relatively objective methods, the diagnosis by ICU physicians based mainly on clinical symptoms of SAE may be somewhat subjective. Hence, missed diagnoses or misdiagnoses might occur. Second, with more than one examiner, errors may be encountered in results interpretation. Moreover, reproducibility and minimizing observers’ variation are crucial concerns in using ONSD sonographic assessment for repeated monitoring. We established a unified standard ONSD measurement method for all our patients to be used by an experienced operator (our two examiners had the same level of experience on ocular ultrasound and used the same examination technique). Standardizing the examination method could help to maintain consistency and reduce observers’ variation. Perfectly, such technical factors standardization may help perform future well-designed clinical studies to evaluate ultrasonographic ONSD benefits.

## Conclusions

In conclusion, patients with SAE had a wider ONSD, and ultrasonographic assessment of ONSD could be a reliable, noninvasive, rapid, and easily accessible objective screening method helpful in early diagnosis of these patients, with a cutoff level > 5.2 mm. A multimodal approach based on clinical examination, neuroimaging, and the bedside ultrasonographic ONSD assessment may play a crucial role in early diagnosis and management of SAE and optimizing patient outcomes.

## Data Availability

The data sets used and/or analyzed during the current study are available from the corresponding author upon reasonable request.

## Clinical Trial Registration

ClinicalTrials.gov identifier NCT05849831.
